# TRANSITIONS IN SOCIAL SUPPORT EXCHANGE PROFILES OVER TIME AMONG OLDER ADULTS

**DOI:** 10.1093/geroni/igac059.1218

**Published:** 2022-12-20

**Authors:** Pildoo Sung, Rahul Malhotra, Grand Cheng, Angelique Chan

**Affiliations:** Centre for Ageing Research and Education, Duke-NUS Medical School, Singapore, Singapore; Duke-NUS Medical School, Singapore, Singapore; National University of Singapore, Singapore, Singapore; Duke-NUS Medical School, Singapore, Singapore

## Abstract

Despite increasing interest in social support exchanges among older adults, little is known about the interplay between giving and receiving social support, how social support exchanges change over time, and factors associated with such change. Using data on 1,305 older Singaporeans participating in two waves of a national, longitudinal survey conducted in 2016-2017 and 2019, we investigated (1) distinct social support exchange profiles that comprise different types of giving and receiving social support, (2) transitions in social support exchange profiles over time, and (3) association of sociodemographic characteristics and health status with such transitions. Gender-stratified random intercept latent transition analysis (RI-LTA) produced three main findings. First, we identified four social support exchange profiles—multi-exchange, provider, receiver, and low exchange—for both males and females at both waves, although the distribution of profiles varied by gender and waves. Second, males were more likely to transition from the multi-exchange profile to other types, whereas females were relatively more likely to transition into the multi-exchange profile over time. Third, among males, those older, of ethnic minority, unmarried, employed, and with depressive symptoms were more likely to transition into the receiver profile from other types. Females who were younger, of ethnic majority, married, and less educated were more likely to transition into the multi-exchange type from low or receiver profiles. The findings capture the temporal dynamics in social support exchange profiles and their gendered characteristics. Policy interventions should focus on older adults who lack social support exchanges and those who lose social support exchanges over time.

